# Rare metastatic sites of renal cell carcinoma: a case series

**DOI:** 10.11604/pamj.2022.42.26.33578

**Published:** 2022-05-11

**Authors:** Anurag Singla, Umesh Sharma, Arun Makkar, Pirzada Faisal Masood, Hemant Kumar Goel, Rajeev Sood, Arvind Ahuja, Ravikant Singh

**Affiliations:** 1 Department of Urology and Renal Transplant, Atal Bihari Vajpayee Institute of Medical Sciences and Dr. Ram Manohar Lohia Hospital, New Delhi, India

**Keywords:** Renal cell carcinoma, scalp, jaw, breast, parotid, metastasis

## Abstract

Renal Cell Carcinoma (RCC) is a lethal cancer with a propensity for wide metastasis. The patterns of metastases are not clearly defined, and patients can present with metastasis to unusual sites at the time of diagnosis of the primary tumor or years after radical nephrectomy. Individual diagnostic and surgical approaches are needed to achieve complete resection with disease-free margins, even in the presence of unusual metastatic sites, multifocality, or history of previous metastasectomy. This provides palliation for symptoms and an opportunity for meaningful disease-free and overall survival. Here we present five cases of RCC with metastasis to unusual sites (scalp, jaw, forearm, parotid, breast, and skeletal muscle). Patients were treated with cytoreductive nephrectomy and/or metastasectomy wherever feasible and/or targeted therapy. In conclusion, a high index of suspicion and accurate diagnosis is important as metastasis to unusual sites presents with atypical manifestations and may masquerade as local pathology, misleading the clinician and directly affecting prognosis and survival.

## Introduction

Renal Cell Carcinoma (RCC) comprises about 90% of all malignant renal tumors and is characterised by potential metastatic extension to lymph nodes, lungs, liver, opposite kidney, adrenal glands, brain, and bones [1]. The widespread use of radiologic imaging has resulted in a significant increase in the incidental detection of kidney tumors [1,2]. Renal cancers have a strong tendency to metastasize with occasionally unpredictable patterns of spread to orbit, parotid gland, paranasal sinuses, tongue, tonsils, thyroid, heart, skin, and muscles [1]. Late metastases from RCC, even decades after potentially curative surgical excision of the primary tumor, have been reported in the literature, with distant metastasis eventually developing in about one out of three patients with RCC [3]. Here we present five cases of RCC with unusual metastasis to the scalp, forearm, breast, skeletal muscle, and parotid gland along with their clinical and management profiles.

## Methods

This study was carried in the Department of Urology and Renal Transplant, ABVIMS and Dr Ram Manohar Lohia Hospital, New Delhi, India. Prospective review of five cases was done who developed unusual metastasis. The clinical characteristics, management, and outcomes of patients were summarized in Table 1. The collection of follow-up data was carried out from physical and electronic medical records.

**Table 1:** the brief description of the cases series

No	Age	Sex	Unusual metastasis	Other metastatic sites	Treatment	Time to metastasis (Year)	Survival (Year)
1	53	M	Rt upper jaw, scalp	Lungs, vertebral column, Lt femur, Lt acetabulum, ribs, Rt clavicle	WLE, targeted therapy, immunotherapy	1.5	3
2	54	M	Rt forearm	-	WLE, targeted therapy	-	3
3	55	F	Rt breast	Rt lung	WLE, targeted therapy	Synchronous	alive
4	54	M	Rt trapezius	Diaphragm, Rt psoas major, Lt erector spinae, Lt gluteus medius	Targeted therapy	4	alive
5	59	M	Rt parotid gland	-	Targeted therapy, RT	4	alive

M: male, F: female, WLE: wide local excision, RT: radiation therapy, Rt: right, Lt: left

## Results

The mean age was 55 years with a range of 53-59 years. All patients were diagnosed cases of RCC on histopathological examination (HPE). They were followed prospectively over years during which they presented with these unusual rare metastasis. One case had synchronous metastasis to the right breast and the right lung. The cases were managed by a multimodal approach involving surgery, targeted therapy, immunotherapy and radiotherapy. The following is the detailed description of each case.

**Figure 1:**
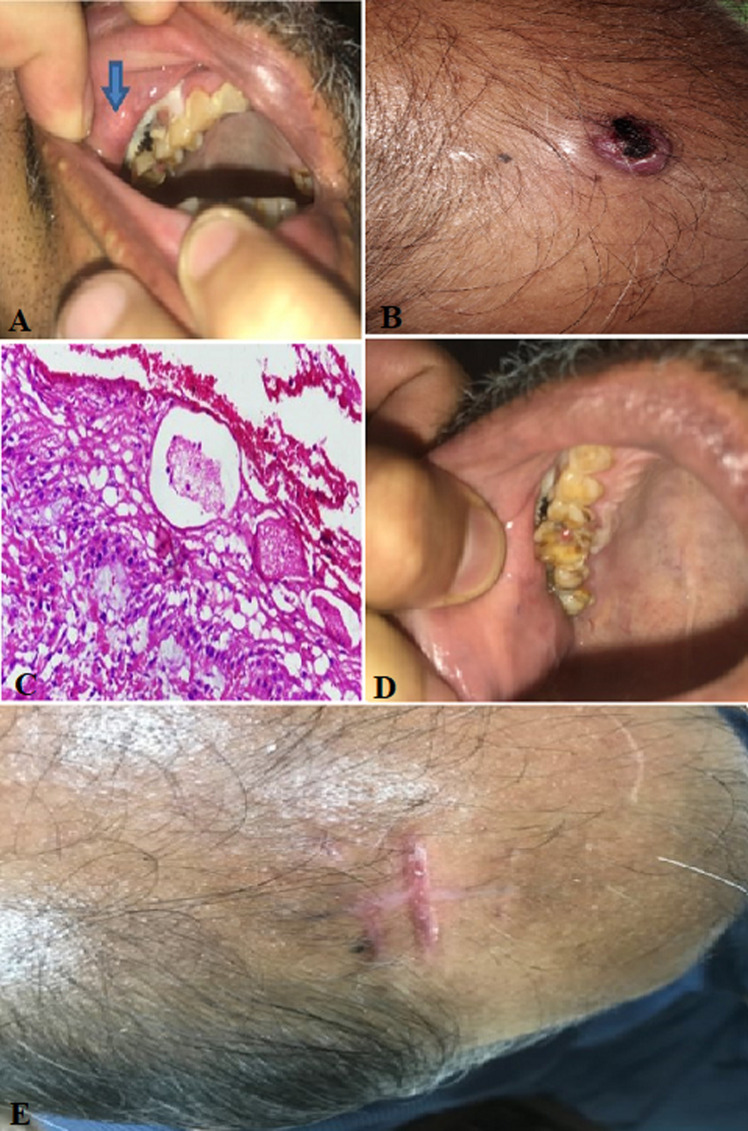
A) upper jaw nodular lesion (arrow); B) ulcerated lesion in the scalp; C) H&E-stained slide at low power (x100) showing a subepithelial sarcomatoid renal cell carcinoma causing focal ulceration of overlying skin; D) post resection healthy jaw lesion; E) post resection healthy scalp lesion

**Case 1:** a 53-year-old male presented with weight loss and a right flank palpable mass for the last six months. Contrast-enhanced computed tomography (CECT) revealed a right renal mass (9 x 7 x 8 cm) in the upper and mid pole infiltrating Gerota fascia with lung metastasis (11 mm at the hilum). Cytoreductive nephrectomy was done and histopathological examination revealed clear cell carcinoma with sarcomatoid changes (<10%). The patient was treated with Sunitinib. On 15 months of follow-up, the patient developed a small 0.5 x 0.5 cm, hard, nodular, well-defined lesion in the right upper jaw. The excisional biopsy revealed metastatic RCC with sarcomatoid features (Figure 1A). Because of disease progression, Axitinib was added to his therapy. The disease continued to progress, and at 17 months, the patient had an ulcerated lesion (0.5 x 1 cm) on the scalp (Figure 1B). The surgical excision was performed and the pathology report revealed metastatic RCC with sarcomatoid features (Figure 1C). After a multidisciplinary tumor board discussion, Nivolumab was added in 2 weekly cycles. Follow-up after two years showed regression of the disease with post-healthy jaw and scalp lesions (Figure 1D, E).

**Figure 2:**
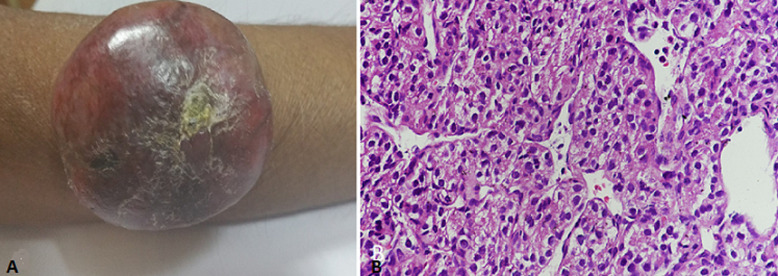
A) right upper forearm lesion; B) high power magnification (x400) showing alveolar nests of tumor cells separated by thin fibrovascular septa. Individual tumor cells are round to polygonal with well-defined borders, clear to eosinophilic cytoplasm and ovoid hyperchromatic nuclei

**Case 2:** a 54-year-old male case of Von Hippel Lindau Syndrome (VHL) and bilateral renal tumors presented with gross haematuria for the last six months. Contrast-enhanced computed tomography (CECT) of abdomen and chest revealed bilateral renal masses with inferior vena cava tumor thrombosis from the right renal masses. The kidney biopsy revealed clear cell carcinoma, and the patient was put on Sunitinib. After two years, the patient developed a lesion over the right upper forearm measuring 5 x 3 cm, which was globular, solid, non-ulcerated with rounded margins (Figure 2A). The excisional biopsy revealed metastatic RCC (Figure 2B). The patient was planned to switch to Axitinib, but he was lost to follow up. On review telephonically, the patient had expired with a survival of 3 years.

**Figure 3:**
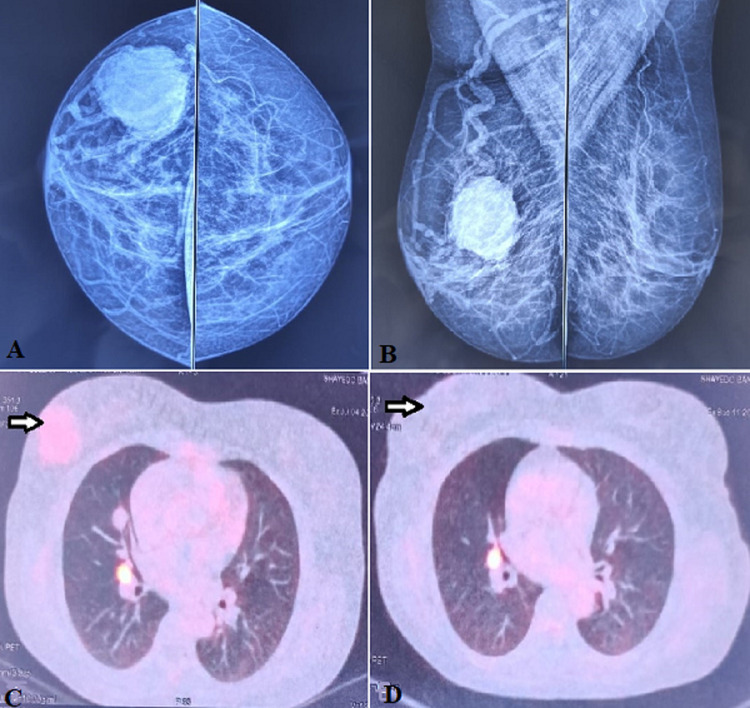
A, B) craniocaudal view and mediolateral oblique mammography views breast; C): PET-CT axial film at breast level showing increased uptake in the right breast (arrow); D) post excision PET-CT showing no lesion in the right breast (arrow)

**Case 3:** a 55-year-old female presented with a right breast lump for two years. On physical examination, a 3 x 3 cm lump was palpable in the right lower quadrant of the right breast. Ultrasonography (USG) revealed a right renal mass and a lesion in the right breast at the 3 o'clock position with no axillary lymphadenopathy. Mammography revealed a hypoechoic irregular mass with indistinct margins, a heterogeneous internal echo pattern, and non-parallel orientation suggestive of a grade IV lesion of the British Imaging-Reporting and Data System (Figure 3A) [4]. Positron emission tomography (PET) scan revealed a large enhancing exophytic mass lesion in the right kidney (16 x 12 x 10 cm), a solitary right breast lesion (3 x 3 cm), and right lung lesion (10 x 11 mm) (Figure 3B). The breast lesion´s fine needle aspiration cytology (FNAC) was inconclusive; thus, a Trucut biopsy was performed and revealed clear cell carcinoma. The patient underwent cytoreductive nephrectomy with right breast lumpectomy. Thoracic surgeon consultation was taken for the excision of the lung nodule but it was not amenable to resection because of its hilar location. Thus, the patient was started on Sunitinib and was kept under surveillance. After three years, follow-up imaging showed stable disease (Figure 3C).

**Figure 4:**
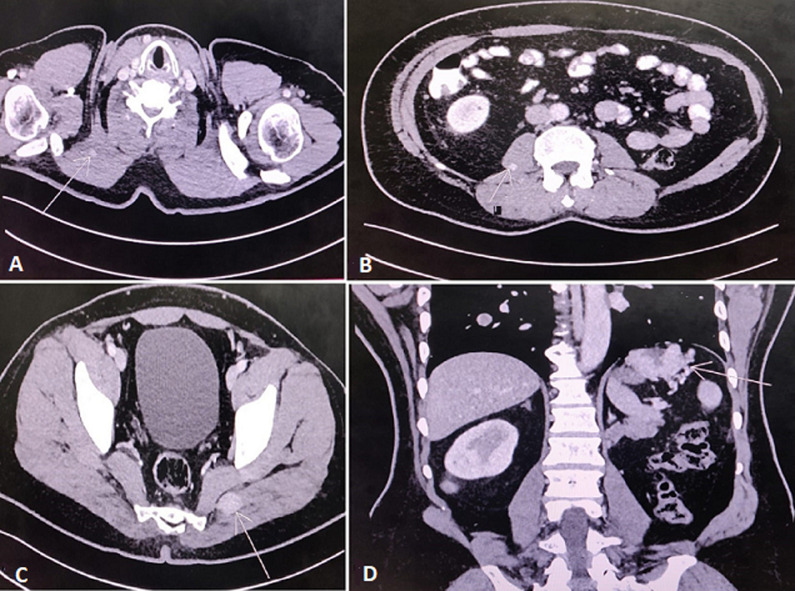
A) CT scan showing metastasis to the right trapezius muscle (arrow); B) metastasis to the right psoas (arrow); C) metastasis to left gluteus medius (arrow); D) metastasis to left hemidiaphragm (arrow)

**Case 4:** a 54-year-old male presented with gross haematuria for the last six months. On radiologic evaluation, he was found to have a large left renal mass. Left radical nephrectomy was performed with HPE-clear cell carcinoma. The patient was followed up with regular imaging until after four years when he presented with generalised body pain, which on evaluation revealed RCC metastasis to the right trapezius muscle (Figure 4A), right psoas muscle (Figure 4B), erector spinae, left gluteus medius muscle (Figure 4C), and left hemidiaphragm (Figure 4D). The patient was treated with Sunitinib and follow-up scans showed stable disease.

**Figure 5:**
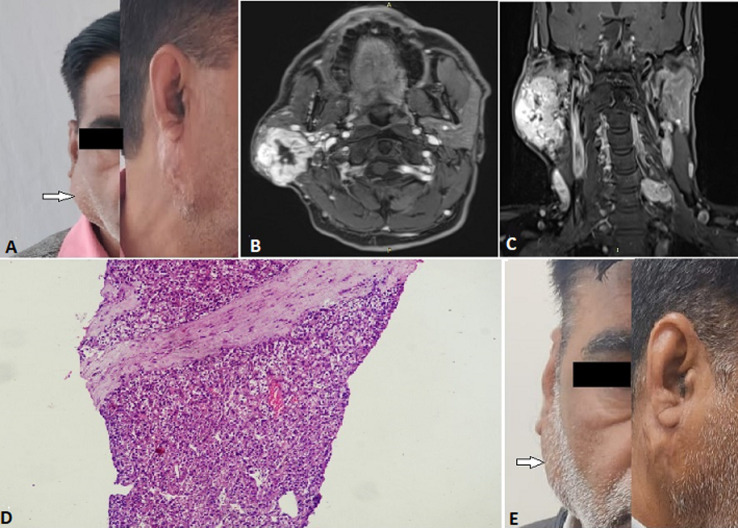
A) right parotid swelling (pre-treatment) (arrow); B) MRI inT1 showing parotid involvement; C) MRI in T2 showing the tumor involvement (coronal view); D) low power magnification (x100): fibro collagenous tissue showing infiltration by a solid tumor arranged in sheets and nests; E) right parotid swelling (post-treatment) (arrow)

**Case 5:** a 59-year-old male operated case of right RCC (clear cell with sarcomatoid features) presented with a swelling in the right preauricular area 4 years after right radical nephrectomy. The clinical examination revealed a 6 x 4 cm mass in the right parotid region (Figure 5A). Contrast-Enhanced Magnetic Resonance Imaging (CEMRI) revealed tumor in the parotid's superficial and deep lobes, abutting the internal jugular vein and sternocleidomastoid muscle with loss of fat planes and engulfment of the adjacent vessels by 180 degrees (Figure 5B, C). Trucut biopsy revealed metastatic clear cell RCC (Figure 5D). Specialist consultation was sought, and the patient was deemed inoperable. He was started on Sunitinib and subjected to radiotherapy to the parotid. The patient received a total dose of 30 Grays in 10 fractions with the Cobalt-60 (Co-60) treatment unit. The patient is on follow-up and shows stable disease (Figure 5E). A brief description of the cases is mentioned in Table 1.

## Discussion

We present rare, unusual metastasis sites of RCC with unpredictable spread patterns to distant skin, jaw, breast, parotid gland, and skeletal muscle. The rates of the presence of skin, jaw, and breast metastasis reported in RCC are 3%, 15%, and 3%, respectively, with occasional reports of parotid and skeletal muscle metastasis [1,5,6]. Usually, RCC primarily metastasizes to lymph nodes, lungs, liver, and bones, while metastasis to other sites is rare [7]. Spread metastasis can be hematogenous, lymphatic, or direct invasion [5]. Cutaneous metastasis of RCC is most common in the scalp, followed by the chest and abdomen, and generally presents at an advanced stage, as was seen in both cases 1 and 2 [8].

As previously reported and seen in our cases 1 and 2, cutaneous metastasis in RCC is predominant in males [8,9]. The time interval to skin metastasis varies from 10 months to 60 months depending upon the tumor stage, with a longer interval for stage I (an average of 51 months) and shorter for higher stages [10]. Cutaneous metastasis from RCC usually presents as a solitary, shiny skin lesion that is red-to-purple in color, and some can be plaque-like or nodular. They can be confused with haemangiomas, pyogenic granulomas, Kaposi's sarcoma, and cutaneous cysts [10]. Right forearm skin metastasis in case 2 was globular, solid, non-ulcerated with rounded margins and red to purple, while in case 1, the scalp metastasis resembled a solitary, ulcerated lesion. Both lesions were managed by wide local excision, similar to that described in previously published articles [7]. Systemic therapy for first-line metastatic cancer RCC is based on Tyrosine Kinases Inhibitors (TKIs), which have demonstrated a significant increase in the chances of survival when compared to other medical therapies (interferon or interleukins). The most commonly used treatments for patients with a good or intermediate prognosis are Sunitinib and Pazopanib. The recommendation to use Temsirolimus or TKIs in patients with a poor prognosis is unclear, and the best supportive care may be an option for the same patients [11,12]. Additionally, Axitinib is currently approved to treat metastatic RCC after failure of prior treatment with Sunitinib or a cytokine. A similar treatment protocol was performed on our patients [13].

Presentation of jaw metastasis is varied, with swelling, pain, paraesthesia, numbness, and a sensation of thickening of the lips being common ones. The mandible is the most commonly affected jaw bone, and the temporomandibular joint the least. Maxillae is affected in 1/7^th^ of jaw metastasis [14]. The treatment options includes: surgical resection, chemotherapy, radiation therapy, or a combination. This preserves oral function and reduces pain and tumor-associated morbidity [15]. In our case 1, the right upper jaw was involved and managed with wide local excision. The prognosis of oral metastatic tumors is poor, with a mean survival of 6-7 months [16]. Our patient succumbed after 21 months post excision of the jaw metastasis.

Breast metastasis from extramammary malignant neoplasms is rare, accounting for 0.3-2.7% of all breast tumors. It can be solitary or multiple, synchronous or metachronous, with a better prognosis if presented later [17]. Our case was synchronous with the initial presentation of a solitary breast lump and a renal mass. Clinically, breast metastasis presents as painless, discrete mobile masses with rapid growth and without involvement of overlying skin and axillary lymph nodes, as seen in our case [18]. Mammogram shows an irregular mass with indistinct margins, with a heterogeneous internal echo pattern, non-parallel orientation, and usually do not show speculation or microcalcifications [19]. Ultrasonography shows a hypoechoic, round, or oval-shaped lesion with posterior acoustic enhancement and no posterior attenuation [19]. Similar findings were reported in our case on mammogram and breast USG, respectively.

The treatment of breast metastasis is different from breast cancer treatment as the skin is not involved, and the axillary node involvement is variable [18]. Chemotherapy is not beneficial as they are chemo resistant. Additionally, targeted therapy has been tried, but there is a paucity of literature [20]. Surgical resection of solitary breast metastasis is associated with better survival and outcomes, and aggressive surgical resection of primary and metastatic lesions is justified [21]. Alzaraa *et al*. reported a case of RCC with breast metastasis treated with surgical excision with a survival of 17 months [22]. We have also treated our case on similar lines with right breast metastasectomy, and the patient is alive till now.

Skeletal muscle metastasis from RCC occurs in 0.4% of patients and is described only in a few case reports. They must be differentiated from primary soft-tissue tumors with open or needle biopsy [23]. MRI features of metastatic RCC to the skeletal muscle may show high-signal intensity on T1- and T2-weighted MRI. Safadi *et al*. reported a case of RCC with metastasis to the erector spinae muscle treated with radical nephrectomy and wide resection of the metastasis [24]. In case 4, the resection was impossible due to multiple muscles and hemidiaphragm involvement. In another case, a 73-year-old female presented with late skeletal muscle metastases from a clear-type renal cell carcinoma eight years after total nephrectomy. The metastases were located in the right femoral quadriceps, sartorius muscle, and adductor magnus muscle. The patient was treated with complete surgical resection with a wide margin, which was performed for all lesions [24]. In another case, a 48-year-old man presented with multiple metastases in his skeletal muscles four years after right radical nephrectomy was carried out for RCC. The tumors are located in the right psoas, paravertebral, and gluteus medius muscles [25].

In the case of musculoskeletal metastasis, the 5-year survival rate for complete metastasectomy in patients with multiple non-lung metastases was 32.5% with complete resection versus 12.4% without complete resection. There is also a potential benefit from the integration of metastasectomy and immunotherapy, with a median survival rate of 4.7 years and a median time to progression of 1.8 years. In patients treated with systemic combinational targeted and immunotherapy using tyrosine kinase inhibitors, interferon-α, interleukin 2 and 5FU, or vinblastine, the mean progression-free survival was 8.1 months, with overall survival of 16.8 months [26].

Parotid metastasis in RCC is exceptionally uncommon, as most usually arises from head and neck skin malignancy. The interval between the initial diagnosis and parotid metastasis ranges from a few months to 10 years [27]. The swelling is usually insidious in onset. Our patient had insidious, painless swelling in the parotid gland four years after undergoing radical nephrectomy. In the case of RCC with parotid metastasis, treatment is a combination of chemotherapy, immunotherapy, hormone therapy, and radiation therapy, but the results are dismal. Depending upon the extent of parotid involvement, surgical treatment varies from superficial parotidectomy with facial nerve preservation to total parotidectomy with neck dissection [27]. Kaplan *et al*. reported a similar case of parotid metastasis from RCC, that survived for six months and was managed by immunotherapy and radiotherapy [28]. In our case, the parotid gland was massively involved by the tumor with extension into the skull base and was not amenable to surgical resection.

Sarcomatoid differentiation can occur in any RCC subtype, including clear cell, papillary, and chromophobe tumors. They are characterised by spindle cells, are aggressive nature, and have the worst prognosis. Sarcomatoid features are an independent predictor of decreased survival and cannot be determined preoperatively. Computed tomography (CT) features of larger tumor size, an increased frequency of peritumoral neovascularity, and global heterogeneity (texture analysis) in the tumor is suggestive of sarcomatoid differentiation [29]. Radical nephrectomies in these cases are technically challenging, and extended lymph node dissection is advised when there are sarcomatoid features on imaging at the time of surgery. Two of our cases had clear cell histopathology with sarcomatoid differentiation. They were larger in size and had heterogenous texture, and were diagnosed after cytoreductive/ radical nephrectomy, similar to the published literature.

Renal cell carcinoma is generally resistant to radiation, traditional cytotoxic drugs and hormonal therapy. Advances in the understanding of RCC biology have resulted in the successful development of targeted therapy for RCC [20]. Patients with limited metastasis can be taken for metastasectomy. Symptomatic bone metastases can be dealt with stereotactic radiation therapy, while brain metastases can be managed surgically or with whole-brain radiation therapy prior to systemic therapy. Surgical resection offers a higher survival advantage in cases of solitary metastasis than no resection [20]. As indicated, we managed our cases with radical or cytoreductive nephrectomy, metastasectomy, targeted therapy, immunotherapy, and radiotherapy.

## Conclusion

Patients with RCC with metastasis to unusual sites are usually have advanced cancers with dismal prognoses. The presentation can be synchronous or metachronous. An individualized, multimodality approach is needed to tackle these complex cases. The surgical approach should be used wherever feasible to achieve complete resection of metastasis with disease-free margins. These strategies provide palliation of symptoms, an opportunity for meaningful disease-free, and overall survival.

### What is known about this topic

- The presence of unusual latent metastasis is characteristic of renal cell carcinoma and may manifest more than years after nephrectomy or at the time of diagnosis of the primary tumor;
- Renal cell carcinoma may metastasis to unusual sites such as scalp, jaw, forearm, parotid, breast, and skeletal muscle.

### What this study adds

- In patients with renal cell carcinoma history, all new lesions should be given attention, even where the primary tumor has been treated;
- History of previous renal cell carcinoma and a high index of suspicion is essential in the proper management of such cases;
- The treatment plan should always be multidisciplinary.
